# Physician Gender and Patient Perceptions of Interpersonal and Technical Skills in Online Reviews

**DOI:** 10.1001/jamanetworkopen.2024.60018

**Published:** 2025-02-14

**Authors:** Farrah Madanay, M. Kate Bundorf, Peter A. Ubel

**Affiliations:** 1 Sanford School of Public Policy, Duke University, Durham, North Carolina; 2 Center for Bioethics and Social Sciences in Medicine, University of Michigan, Ann Arbor; 3 Fuqua School of Business, Duke University, Durham, North Carolina

## Abstract

**Importance:**

Prior studies have revealed gender differences in workplace assessments of physicians, but little is known about differences by physician gender in patients’ online written reviews.

**Objective:**

To analyze whether patients’ perceptions of their physicians’ interpersonal manner and technical competence differ by physician gender and practicing specialty and are associated with review star ratings.

**Design, Setting, and Participants:**

This cross-sectional study sampled written reviews submitted by patients between October 16, 2015, and May 27, 2020, for physicians across the US from a commercial physician rating and review website. Physicians included primary care physicians (PCPs) listed under family medicine, internal medicine, and pediatrics and surgeons listed under general surgery; orthopedic surgery; and cosmetic, plastic, and reconstructive surgery. Hand-coded reviews were used to fine-tune a natural language processing algorithm to classify all reviews for the presence and valence of patients’ comments of physicians’ interpersonal manner and technical competence. Statistical analyses were performed from July 2022 to December 2024.

**Exposure:**

Female or male physician gender.

**Main Outcomes and Measures:**

Outcomes included the presence and valence of interpersonal manner and technical competence comments and receipt of high star ratings. Multilevel logistic regressions analyzed differences by female or male physician gender in interpersonal manner and technical competence comments and whether those comments were associated with review star ratings.

**Results:**

The analysis included 345 053 written reviews of 167 150 physicians (mean [SD] age, 55.16 [11.40] years); 60 060 physicians (35.9%) were female, and 36 132 (21.6%) were surgeons. Female physicians overall had higher odds than males of receiving any (odds ratio [OR], 1.19; 95% CI, 1.16-1.22) or negative (OR, 1.22; 95% CI, 1.18-1.26) patient comments for their interpersonal manner. Among PCPs, females had higher odds than males of receiving a negative comment for interpersonal manner (OR, 1.22; 95% CI, 1.18-1.27) and, when receiving that negative comment, had disproportionately lower odds of receiving a high star rating (OR, 0.62; 95% CI, 0.53-0.73). Female physicians overall (OR, 1.09; 95% CI, 1.05-1.13) and female PCPs (OR, 1.08; 95% CI, 1.04-1.13) had higher odds than their male counterparts of receiving a negative comment for their technical competence. When receiving a negative comment for technical competence, both female PCPs (OR, 0.60; 95% CI, 0.50-0.73) and female surgeons (OR, 0.67; 95% CI, 0.50-0.89) had disproportionately lower odds of receiving a high star rating compared with their male counterparts. Female PCPs also had lower odds than male PCPs of receiving a high star rating when receiving a positive comment for technical competence (OR, 0.82; 95% CI, 0.70-0.96).

**Conclusions and Relevance:**

In this cross-sectional study of online written reviews, female and male physician gender were differently associated with patients’ perceptions of their physicians’ interpersonal manner and technical competence. The findings suggest that patients harbored negative gender biases about the interpersonal manner of female physicians, especially female PCPs, and also assessed disproportionate penalties related to technical competence for both female PCPs and female surgeons.

## Key Points

**Question:**

Do patients perceive physicians differently in online written reviews based on female or male physician gender?

**Findings:**

In this cross-sectional study of 345 053 online reviews of 167 150 physicians, female physicians had higher odds than male physicians of receiving patient comments about their interpersonal manner. In some cases, female primary care physicians and surgeons either had higher odds of being penalized or lower odds of being rewarded by patients in star ratings for their interpersonal manner or technical competence.

**Meaning:**

The findings suggest that differences by physician gender exist in patients’ online reviews, vary by practicing specialty, and disproportionately penalize female physicians in star ratings.

## Teaser

This cross-sectional study investigates whether patient assessments of physicians’ interpersonal manner and technical competence and the star ratings they give in online reviews differ by female or male physician gender.

## Introduction

Patients increasingly use online forums to report the quality of physicians. They not only provide overall ratings (eg, 4 stars) but also post written reviews. These written reviews tend to fall into 2 dimensions: interpersonal manner and technical competence. Interpersonal manner includes patients’ comments about their physicians’ communication, care, and personal engagement.^1,2^ A physician might be described as warm and personable or as frigid and having poor bedside manner. Technical competence encompasses patients’ perceptions of their physicians’ efficiency, skill, and knowledge.^1,2^ A physician might be described as an expert and thorough or as incompetent and sloppy. Research shows that these 2 dimensions undergird patient-centered care^2,3^ and align with the warmth and competence dimensions from social psychology.^4^

Although patients may be better equipped to assess their physicians’ interpersonal manner than their technical competence,^5^ online physician reviews include patients’ comments about both. Prospective patients consider online reviews to be an important factor when choosing a physician,^6^ they value the quality and helpfulness of written reviews,^7,8^ and they trust the accuracy of online ratings and reviews more than government ratings and traditional patient experience surveys.^6,9,10^ However, because insights from these written reviews are challenging to extract without extensive qualitative analysis or natural language models,^1,11,12^ little is known about the perceptions that patients share about their physicians in online written reviews.

Researchers have documented many types of gender biases that have impacted rates of hiring, promotion, compensation, and scholarship across medical specialties and ranks.^13,14,15,16,17^ Social psychologists have shown that men are expected to be high in competence and low in warmth and women to be high in warmth and low in competence.^4^ These gender stereotypes negatively affect women in performance assessments used for hiring and promotion across occupations,^18,19,20,21,22^ including in medicine.^23,24,25,26^

In this study, we analyzed differences in patients’ written reviews by physician gender using data from a commercial physician review website. We also analyzed whether these gender differences were consistent across physician specialties, including generalists like primary care physicians (PCPs) and proceduralists like surgeons. We used an advanced machine learning algorithm to classify the reviews at scale for the presence and valence of patients’ interpersonal manner and technical competence perceptions.

## Methods

### Data Source

In this cross-sectional study, we collected physician profile, star rating, and written review data from Healthgrades.com^27^ in May 2020. Reviews were submitted between October 16, 2015, and May 27, 2020. Healthgrades.com is one of the largest commercial physician rating and review websites, with profiles for every US physician active on the National Provider Identifier Registry. Patients can choose to leave a star rating of 1 to 5 on a physician’s profile with or without a written (narrative) review of their physician encounter. The study was approved by the Duke University institutional review board, with consent waived because the research involved a secondary analysis of identifiable private information that is publicly available. This study followed the Strengthening the Reporting of Observational Studies in Epidemiology (STROBE) reporting guideline for cross-sectional studies.

We collected profiles associated with 3 primary care specialties (family medicine, internal medicine, and pediatrics) and 3 surgical specialties (general surgery; orthopedic surgery; and cosmetic, plastic, and reconstructive surgery). All physicians from these 6 specialties who received at least 1 written review between October 16, 2015, and May 27, 2020, were included in the sample. From each profile, we extracted the text of up to 20 of the physician’s most recent written reviews and each review’s accompanying star rating (1-5 stars). If a physician had more than 20 written reviews between 2015 and 2020, we collected the 20 most recent reviews; if a physician had 20 or fewer reviews, we collected all the reviews between 2015 and 2020. We also extracted the physician’s gender, age, and specialty and the state in which the physician’s primary office was located.

### Study Variables

Our key study outcomes were the presence and valence of interpersonal manner and technical competence perceptions for each written review, which we measured using natural language processing techniques. We first purposely sampled 2000 random written reviews for equal representation of PCPs and surgeons, female and male physicians, and low (≤3 stars) and high (≥4 stars) star review ratings. Using qualitative content analysis, we hand coded the 2000 reviews for the presence (any vs none) and valence (positive or negative) of patients’ perceptions of their physicians’ interpersonal manner and technical competence. Four coders (including F.M.) achieved high interrater reliability with a subset of 300 double-coded reviews (Cohen κ range, 0.74-0.85) before proceeding to independently code the remaining reviews, with 10% of reviews being double coded to ensure continued high interrater reliability (Cohen κ range, 0.80–0.92). Interpersonal manner included patients’ perceptions of physicians’ attitude and character, behavior, and communication. Technical competence included patients’ perceptions of physicians’ expertise, treatment approach, and outcomes. A patient’s review could include both interpersonal manner and technical competence perceptions, but each perception was given a single valence (positive, negative, or none).

We used the 2000 hand-coded reviews to fine-tune a pretrained natural language processing algorithm called the Robustly Optimized BERT Pretraining Approach (RoBERTa) to classify the full sample of written reviews for the presence and valence of interpersonal manner and technical competence perceptions.^28^ For both classification models, 1 for interpersonal manner and 1 for technical competence, we tuned the models with 1600 reviews randomly sampled from our hand-coded reviews. We then used a new set of 200 randomly sampled hand-coded reviews to test each model’s fit while further fine-tuning the models. Last, we used our final 200 hand-coded reviews, which were withheld from the training and testing data, to provide unbiased evaluations of each model’s classification performance. Our interpersonal manner and technical competence classification models each demonstrated 90% accuracy with high precision (0.89 and 0.91, respectively), recall (0.90 and 0.90, respectively), and weighted *F*_1_ scores (0.89 and 0.90, respectively). Detailed methods of our classification algorithm are described elsewhere.^29^

Outcomes included 6 binary variables based on the content of the written reviews: (1) any perception of interpersonal manner, (2) positive interpersonal manner, (3) negative interpersonal manner, (4) any perception of technical competence, (5) positive technical competence, and (6) negative technical competence. Any perception took the value of 1 for presence and 0 for absence. Positive perceptions took the value of 1 for positive valence and 0 for negative valence or no perception.

An additional outcome was a binary indicator of a high star review rating. Based on prior literature, the indicator took the value of 1 for a 4- to 5-star written review and 0 for a 1- to 3-star written review.^7,30,31,32^

Our main exposure was physician gender (female or male) as listed in the physician’s profile. The physician review website did not list nonbinary or other genders. We also extracted information on physician specialty (primary care vs surgery), physician age, state where the physician’s primary office was located, year the review was submitted (2015-2020), and written review word count.

### Statistical Analysis

Descriptive statistics were computed with means, SDs, and frequencies. Multilevel logistic regressions were performed using physician review–level data and accounting for dependence of reviews nested within physicians.^33^ Random effects at the physician level were included to account for clustering of patients’ reviews within physicians. Two-sided *P* < .05 was considered significant.

To test whether patients perceived female and male physicians differently, we estimated models of physicians overall and separately by specialty (primary care vs surgery). For each sample, we ran 6 models corresponding to the 6 perception outcomes. The independent variable of primary interest was an indicator of female physician gender. We reported the odds ratios (ORs) for each outcome for female compared with male physicians.

To test whether patients rated female and male physicians differently based on perception dimension, we estimated models separately by specialty with an indicator of a high star review rating as the dependent variable. Independent variables included an indicator of female physician gender, indicators of positive and negative interpersonal manner and technical competence, and interactions between female physician gender and each perception indicator. The outcomes of primary interest were the interactions between the indicator of female physician gender and patient perception dimensions. We reported the ORs of high star ratings received by female physicians compared with male physicians when they received positive or negative patient comments about their interpersonal manner or technical competence.

All regressions controlled for physician age category, an indicator of the state where the physician’s primary office was located, an indicator of review submission year, and the review word count. Regressions estimated with the full sample of physicians additionally controlled for an indicator of PCP physician specialty. Statistical analyses were performed from July 2022 to December 2024 using Stata, version 17.0 (StataCorp LLC).^34^

## Results

### Characteristics of Study Sample

We collected a total of 446 475 physician profiles. The final sample included 345 053 online written reviews received by 167 150 US physicians. Physicians’ mean (SD) age was 55.16 (11.40) years; 19.8% were aged 19 to 45 years; 22.0%, 46 to 53 years; 23.6%, 54 to 62 years; and 25.4%, 63 years or older (9.2% were missing age data). A total of 60 060 (35.9%) were female, and 107 090 (64.1%) were male; 36 132 (21.6%) were surgeons and 131 018 (78.4%) were PCPs. We excluded physicians who did not receive at least 1 written review. Most written reviews (77.3%) had 5-star ratings, followed by 1-star ratings (18.5%) (eFigure in Supplement 1). There were more PCPs than surgeons in the sample (78.4% vs 21.6%); however, because surgeons on average received more written reviews, the 2 specialties received nearly equal shares of reviews (49.1% received by PCPs vs 50.9% received by surgeons) (Table).

**Table.** Physician-Level and Review-Level Characteristics

Characteristic	PCPs		Surgeons		Total
	Female	Male	Female	Male	
**Physician-level characteristics**					
Physicians, No. (%)	55 814 (33.4)	75 204 (45.0)	4246 (2.5)	31 886 (19.1)	167 150 (100)
Written reviews per physician, mean (SD)^a^	1.36 (1.46)	1.24 (1.21)	3.24 (4.62)	5.08 (5.99)	2.06 (3.32)
Physician age, mean (SD), y^b^	51.10 (10.19)	57.55 (11.42)	50.45 (9.72)	56.84 (11.43)	55.16 (11.40)
**Review-level characteristics**					
Written reviews, No. (%)^a^	75 941 (22.0)	93 326 (27.0)	13 769 (4.0)	162 017 (47.0)	345 053 (100)
Written review rating, mean (SD)	3.94 (1.72)	4.01 (1.68)	4.38 (1.41)	4.37 (1.42)	4.18 (1.58)

Abbreviation: PCP, primary care physician.

^a^ A maximum of 20 written reviews per physician were scraped, even though some physicians in the dataset could have had more than 20 written reviews on the physician review website.

^b^ Physician age was missing for 15 406 physicians (9.2%) (female PCPs: n = 6702; male PCPs: n = 5349; female surgeons: n = 658; male surgeons: n = 2697). Age was conditional on nonmissing responses.

### Characteristics of Written Reviews

**Figure 1.**
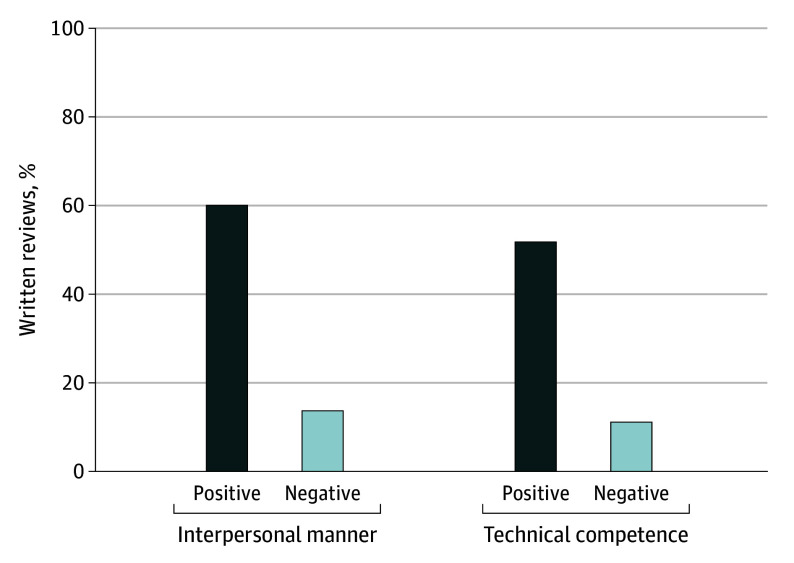
Percentage of Written Reviews With Positive and Negative Interpersonal Manner and Technical Competence Perceptions

The mean (SD) word count of written reviews was 49.8 (35.2). Of all written reviews, 74.0% mentioned interpersonal manner, with more positive than negative interpersonal manner perceptions. Similarly, 62.9% of reviews mentioned technical competence, with more positive than negative technical competence perceptions (Figure 1).

### Patients’ Perceptions of Physicians

#### Overall

Overall, female physicians compared with male physicians had increased odds of receiving an interpersonal manner comment (OR, 1.19; 95% CI, 1.16-1.22; *P* < .001) and of receiving a negative patient comment for their interpersonal manner (OR, 1.22; 95% CI, 1.18-1.26; *P* < .001). Female and male physicians had equal odds of receiving a technical competence comment (OR, 1.00; 95% CI, 0.98-1.02; *P* = .78), but female physicians had higher odds of receiving a negative patient comment for their technical competence (OR, 1.09; 95% CI, 1.05-1.13; *P* < .001) and lower odds of receiving a positive technical competence comment (OR, 0.97; 95% CI, 0.95-0.99; *P* = .02). eTable 1 in Supplement 1 reports ORs, estimated probabilities, and physician-level random effects for female compared with male physicians overall.

#### Primary Care Physicians

Female PCPs compared with male PCPs had higher odds of receiving an interpersonal manner comment (OR, 1.13; 95% CI, 1.10-1.16; *P* < .001) and of receiving a negative patient comment for their interpersonal manner (OR, 1.22; 95% CI, 1.18-1.27; *P* < .001). Female and male PCPs had equal odds of receiving a technical competence comment (OR, 1.00; 95% CI, 0.98-1.03; *P* = .73). However, female PCPs had higher odds of receiving a negative patient comment for their technical competence (OR, 1.08; 95% CI, 1.04-1.13; *P* < .001). eTable 2 in Supplement 1 reports ORs, estimated probabilities, and physician-level random effects for female compared with male PCPs.

#### Surgeons

**Figure 2.**
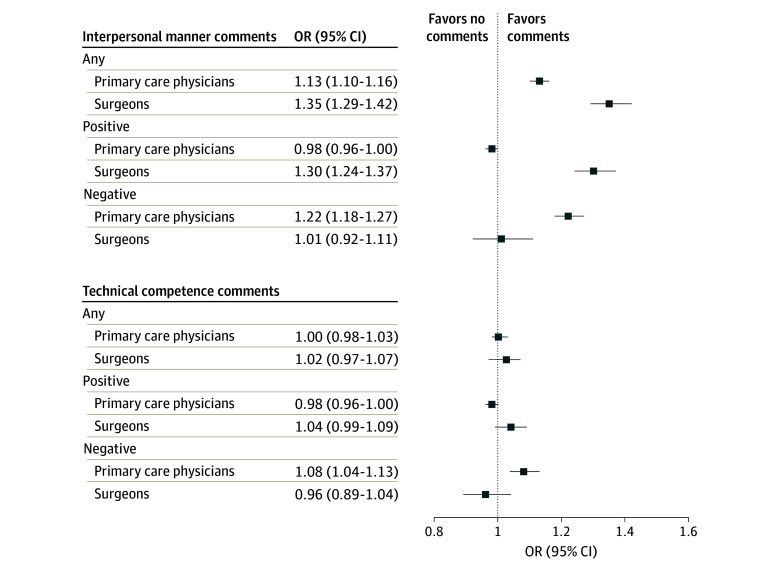
Odds of Receiving Patient Comments in Written Reviews for Female Compared With Male Physicians, by Specialty OR indicates odds ratio.

Female surgeons compared with male surgeons had higher odds of receiving an interpersonal manner comment (OR, 1.35; 95% CI, 1.29-1.42; *P* < .001) and of receiving a positive patient comment for their interpersonal manner (OR, 1.30; 95% CI, 1.24-1.37; *P* < .001). Female and male surgeons had equal odds of receiving a technical competence comment (OR, 1.02; 95% CI, 0.97-1.07; *P* = .42). eTable 3 in Supplement 1 reports ORs, estimated probabilities, and physician-level random effects for female and male surgeons. Figure 2 shows ORs and 95% CIs for patients’ interpersonal manner and technical competence comments received by female compared with male PCPs and surgeons in online written reviews.

### Association Between High Star Ratings and Patient Perceptions

#### Primary Care Physicians

Female and male PCPs who received a positive patient comment for their interpersonal manner had equal odds of receiving a high star rating (OR, 1.01; 95% CI, 0.91-1.14; *P* = .81). However, among PCPs who received a positive patient comment for their technical competence, female PCPs had decreased odds of receiving a high star rating compared with male PCPs (OR, 0.82; 95% CI, 0.70-0.96; *P* = .01).

Among PCPs who received a negative patient comment for their interpersonal manner, female PCPs had decreased odds of receiving a high star rating compared with male PCPs (OR, 0.62; 95% CI, 0.53-0.73; *P* < .001). Similarly, among PCPs who received a negative patient comment for their technical competence, female PCPs had decreased odds of receiving a high star rating compared with male PCPs (OR, 0.60; 95% CI, 0.50-0.73; *P* < .001). eTable 4 in Supplement 1 reports ORs, estimated probabilities, and physician-level random effects for female and male PCPs receiving high star ratings when patients commented on their interpersonal manner or technical competence.

#### Surgeons

Among surgeons who received a positive patient comment for their interpersonal manner, female and male surgeons had equal odds of receiving a high star rating (OR, 1.09; 95% CI, 0.83-1.43; *P* = .54). Likewise, among surgeons who received a positive patient comment for technical competence, female and male surgeons had equal odds of receiving a high star rating (OR, 1.06; 95% CI, 0.77-1.45; *P* = .72).

**Figure 3.**
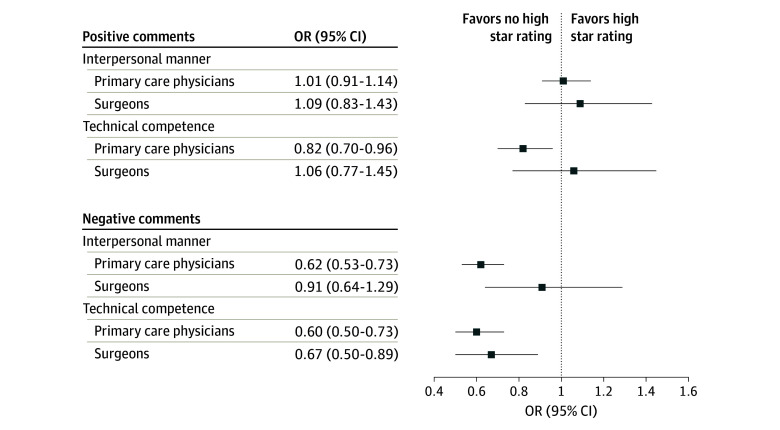
Odds of Receiving High Star Ratings for Female Compared With Male Physicians in Patients’ Comments in Written Reviews, by Specialty OR indicates odds ratio.

Among surgeons who received a negative patient comment for their interpersonal manner, female and male surgeons had equal odds of receiving a high star rating (OR, 0.91; 95% CI, 0.64-1.29; *P* = .58). However, among surgeons who received a negative patient comment for their technical competence, female surgeons had decreased odds of receiving a high star rating compared with male surgeons (OR, 0.67; 95% CI, 0.50-0.89; *P* = .01). eTable 5 in Supplement 1 reports ORs, estimated probabilities, and physician-level random effects for female and male surgeons receiving high star ratings when patients commented on their interpersonal manner or technical competence. Figure 3 shows ORs and 95% CIs for high star ratings received by female compared with male PCPs and surgeons when patients commented on their interpersonal manner or technical competence.

## Discussion

In this cross-sectional study of online written reviews, female physicians overall were more likely than male physicians to receive any patient comments about their interpersonal manner and negative patient comments about their interpersonal manner. Female PCPs were especially more likely to receive patient criticism of their interpersonal manner, and when receiving this criticism, they were disproportionately penalized in star ratings compared with male PCPs. This finding suggests that patients harbored negative gender biases about the interpersonal manner of female PCPs.

Female physicians overall and female PCPs, in particular, were more likely than male physicians to receive negative comments about their technical competence. When criticized for their technical competence, both female PCPs and female surgeons were disproportionately penalized in star ratings compared with their male counterparts. Moreover, female PCPs were less likely rewarded in star ratings when receiving patient praise for their technical competence compared with male PCPs. This finding suggests that patients assessed a gender penalty related to technical competence for both female PCPs and female surgeons.

This research corroborates prior studies that found female physicians were more likely to receive interpersonal manner comments.^30,35,36^ However, whether female physicians were more likely to receive patient praise or criticism depended on their specialty. Social psychology research suggests that individuals penalize people whose behaviors violate their gender stereotype.^37^ Female PCPs face 2 compounding interpersonal stereotypes: both women and PCPs are expected to be warm.^4^ Thus, seemingly cold female PCPs may have been particularly salient to patients, who then perceived these female PCPs more harshly than they would male PCPs.^4,37^ Alternatively, female PCPs may have provided objectively worse patient-centered care than their male counterparts. Yet, research has shown that female physicians are more patient centered than males, providing a reason to doubt this argument.^3,38^

Double standards research may explain why female surgeons more likely received praise for their interpersonal manner compared with male surgeons. Although double standards can hinder women’s career advancement, double standards can also advantage women who outperform the standard.^39,40,41,42^ Warm female surgeons may have been especially salient to patients for conforming to their interpersonal gender norm while excelling in a male-dominated, highly technical specialty.

Few prior studies have examined physician gender differences in patients’ perceptions of their physicians’ technical competence. Previous literature found patients were more likely to praise the technical skills of high-rated male physicians^43^ and male surgeons^44^ compared with those of female counterparts. In this study, although male physicians were not more likely to receive patient praise for their technical competence, female physicians overall and female PCPs were more likely to receive criticism. It is possible that female PCPs have objectively worse technical skills than male PCPs. Alternatively, patients may have more easily noticed poor technical performance by female PCPs compared with male PCPs because poor performance confirmed their stereotype bias that women were not as technically competent as men.^45^

In addition, female PCPs and surgeons, in some cases, were more likely than their male counterparts to be penalized by patients in the star ratings for their interpersonal manner or technical competence. Whereas prior research has shown that patients were more likely to give worse ratings to female physicians than male physicians,^30,36,46^ few studies have investigated physician gender differences in how review star ratings correspond with perceptions received in written reviews.^35^ Research on surgeon referrals, however, found gender asymmetries consistent with our findings.^47^ After the same negative outcome, female surgeons incurred more referral and Medicare billing loss compared with male surgeons.^47^ In other words, female surgeons were penalized more than their male counterparts for similarly poor technical competence.

### Limitations

This study has several limitations. First, use of data from a single physician review website limited the generalizability of these findings. Additionally, physicians included in this study were different from those excluded for receiving no written reviews. For example, physicians with reviews received nearly 4 times as many star ratings and lower average ratings than physicians without reviews (eTable 6 in Supplement 1).

Second, this study could not conclude whether the physician gender differences observed reflected objective differences in care, differences in patient expectations of care, or differences in patient sensitivity to how physicians demonstrated interpersonal manner and technical competence. Although researchers have attempted to objectively measure patient-centeredness and perceived communication styles among female and male physicians,^3,48,49^ further research is required to assess the mechanisms behind our findings.

Third, this study did not examine reviews based on other physician characteristics or reviewer characteristics, such as reviewer gender. With commercial online reviews, patients do not need to offer their gender and can write a review anonymously or with a pseudonym, making inferences based on names challenging. Future research should also examine how patients’ perceptions may differ based on physicians’ race and ethnicity, urbanicity, and intersectional identities.

## Conclusions

In this cross-sectional study of online written reviews, female physicians had higher odds than male physicians of receiving star rating penalties from their patients for perceived poor interpersonal manner and technical competence, whereas male physicians received less patient feedback about their interpersonal manner than did female physicians. How patients perceive their physicians in online reviews matters to prospective patients who trust these reviews when choosing a physician^10,11,50^ and to physicians who use these reviews for both feedback and quality improvement.^51^ Potential areas of intervention include increasing awareness among patient reviewers and prospective patients of potential gender differences in physician reviews, retooling survey measures to elicit more gender-balanced perceptions, and providing objective or expert-derived measures of technical competence alongside patients’ written reviews.

## Notes

Supplement 1. **eFigure.** Distribution of Written Review Star Ratings **eTable 1.** Odds Ratios, Estimated Probabilities, and Physician-Level Random Effects of Female vs Male Physicians Receiving Any, Positive, or Negative Interpersonal Manner and Technical Competence Comments **eTable 2.** Odds Ratios, Estimated Probabilities, and Physician-Level Random Effects of Female vs Male PCPs Receiving Any, Positive, or Negative Interpersonal Manner and Technical Competence Comments **eTable 3.** Odds Ratios, Estimated Probabilities, and Physician-Level Random Effects of Female vs Male Surgeons Receiving Any, Positive, or Negative Interpersonal Manner and Technical Competence Comments **eTable 4.** Odds Ratios, Estimated Probabilities, and Physician-Level Random Effects of Female vs Male PCPs Receiving High Star Ratings When Patients Comment on Their Interpersonal Manner and Technical Competence **eTable 5.** Odds Ratios, Estimated Probabilities, and Physician-Level Random Effects of Female vs Male Surgeons Receiving High Star Ratings When Patients Comment on Their Interpersonal Manner and Technical Competence **eTable 6.** Characteristics of Physicians With and Without Written Reviews

Supplement 2.
Data Sharing Statement
