# Error in Key Points

**DOI:** 10.1001/jamanetworkopen.2025.14832

**Published:** 2025-05-02

In the Original Investigation titled “Physician Gender and Patient Perceptions of Interpersonal and Technical Skills in Online Reviews,”^1^ published February 14, 2025, the Key Points Findings were corrected to state that female physicians had higher odds, not lower odds, than male physicians of receiving patient comments about their interpersonal manner. This article has been corrected.^1^
