# Supplementary material for: Physician Gender and Patient Perceptions of Interpersonal and Technical Skills in Online Reviews
Source: JAMA Netw Open. 2025 Feb 14;8(2):e2460018. doi: 10.1001/jamanetworkopen.2024.60018 (PMC11829228; doi:10.1001/jamanetworkopen.2024.60018)
Supplement: Supplement 1. — eFigure. Distribution of Written Review Star Ratings eTable 1. Odds Ratios, Estimated Probabilities, and Physician-Level Random Effects of Female vs Male Physicians Receiving Any, Positive, or Negative Interpersonal Manner and Technical Competence Comments eTable 2. Odds Ratios, Estimated Probabilities, and Physician-Level Random Effects of Female vs Male PCPs Receiving Any, Positive, or Negative Interpersonal Manner and Technical Competence Comments eTable 3. Odds Ratios, Estimated Probabilities, and Physician-Level Random Effects of Female vs Male Surgeons Receiving Any, Positive, or Negative Interpersonal Manner and Technical Competence Comments eTable 4. Odds Ratios, Estimated Probabilities, and Physician-Level Random Effects of Female vs Male PCPs Receiving High Star Ratings When Patients Comment on Their Interpersonal Manner and Technical Competence eTable 5. Odds Ratios, Estimated Probabilities, and Physician-Level Random Effects of Female vs Male Surgeons Receiving High Star Ratings When Patients Comment on Their Interpersonal Manner and Technical Competence eTable 6. Characteristics of Physicians With and Without Written Reviews [file jamanetwopen-e2460018-s001.pdf]

Supplementary Online Content

Madanay F, Bundorf MK, Ubel PA. Physician gender and patient perceptions of interpersonal and technical skills in online reviews. *JAMA Netw Open*. 2025;8(2):e2460018. doi:10.1001/jamanetworkopen.2024.60018

eFigure. Distribution of Written Review Star Ratings

eTable 1. Odds Ratios, Estimated Probabilities, and Physician-Level Random Effects of Female vs Male Physicians Receiving Any, Positive, or Negative Interpersonal Manner and Technical Competence Comments

eTable 2. Odds Ratios, Estimated Probabilities, and Physician-Level Random Effects of Female vs Male PCPs Receiving Any, Positive, or Negative Interpersonal Manner and Technical Competence Comments

eTable 3. Odds Ratios, Estimated Probabilities, and Physician-Level Random Effects of Female vs Male Surgeons Receiving Any, Positive, or Negative Interpersonal Manner and Technical Competence Comments

eTable 4. Odds Ratios, Estimated Probabilities, and Physician-Level Random Effects of Female vs Male PCPs Receiving High-Star Ratings When Patients Comment on Their Interpersonal Manner and Technical Competence

eTable 5. Odds Ratios, Estimated Probabilities, and Physician-Level Random Effects of Female vs Male Surgeons Receiving High-Star Ratings When Patients Comment on Their Interpersonal Manner and Technical Competence

eTable 6. Characteristics of Physicians With and Without Written Reviews

This supplementary material has been provided by the authors to give readers additional information about their work.

© 2025 Madanay F et al. *JAMA Network Open*.

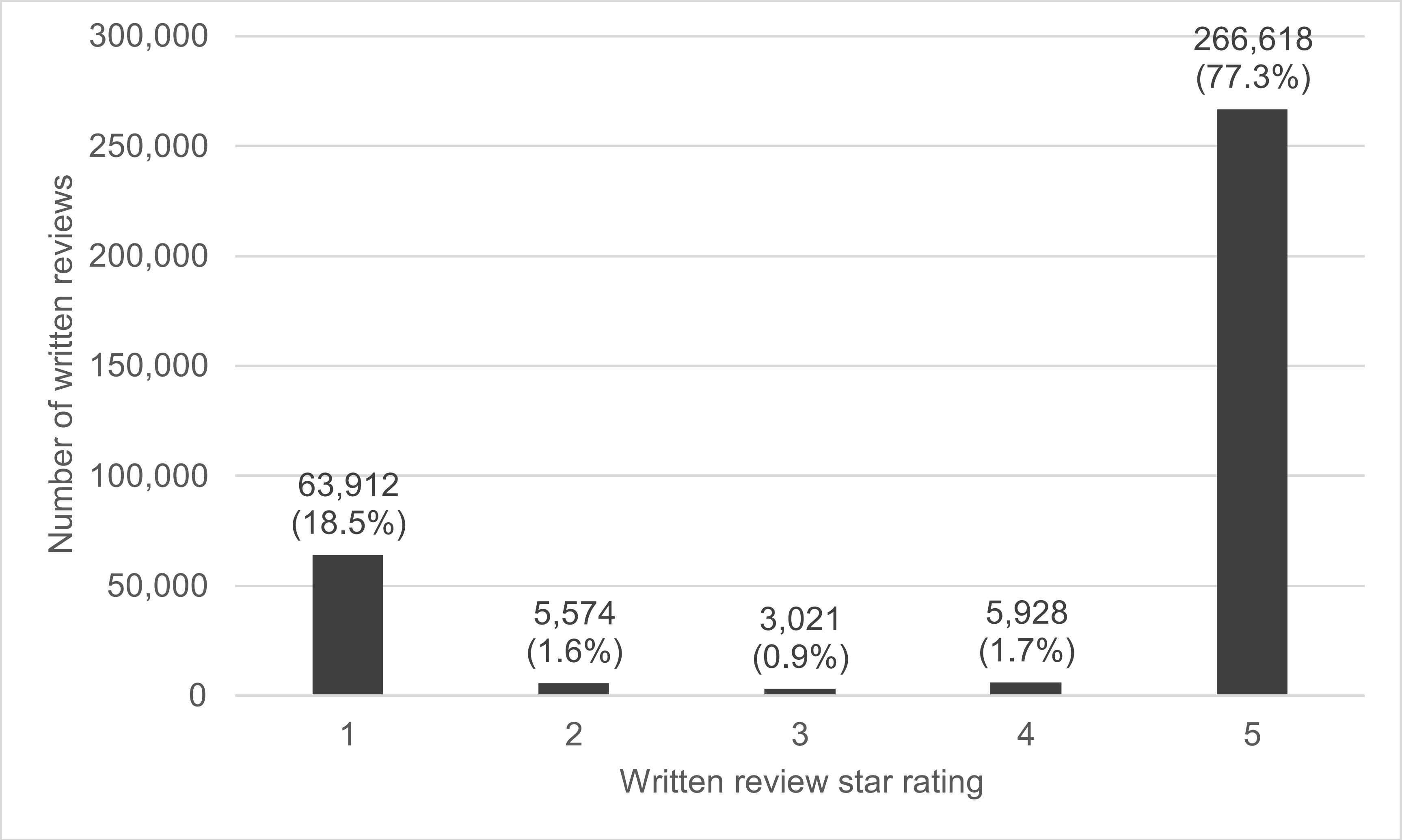

eFigure. Distribution of written review star ratings

© 2025 Madanay F et al. *JAMA Network Open*.

eTable 1. Odds ratios, estimated probabilities, and physician-level random effects of female vs male physicians receiving any, positive, or negative interpersonal manner and technical competence comments

	Any interpersonal manner		Positive interpersonal manner		Negative interpersonal manner		Any technical competence		Positive technical competence		Negative technical competence	
	OR [95% CI]	EP (%)	OR [95% CI]	EP (%)	OR [95% CI]	EP (%)	OR [95% CI]	EP (%)	OR [95% CI]	EP (%)	OR [95% CI]	EP (%)
Physician gender												
Female	1.19*** [1.16, 1.22]	76.3	1.02* [1.00, 1.05]	60.0	1.22*** [1.18, 1.26]	16.0	1.00 [0.98, 1.02]	63.1	0.97* [0.95, 0.99]	50.8	1.09*** [1.05, 1.13]	12.3
Male	1.00	73.3	1.00	59.5	1.00	14.0	1.00	63.0	1.00	51.4	1.00	11.5
Physician-level random effects												
σ^2	0.28 [0.26, 0.30]		0.44 [0.42, 0.46]		1.55 [1.48, 1.62]		0.29 [0.27, 0.31]		0.42 [0.40, 0.44]		1.10 [1.04, 1.16]	
ICC	0.08 [0.07, 0.08]		0.12 [0.11, 0.12]		0.32 [0.31, 0.33]		0.08 [0.08, 0.09]		0.11 [0.11, 0.12]		0.25 [0.24, 0.26]	
LRT (χ^2)	2041.91***		4731.03***		6998.77***		1671.55***		4135.56***		3595.65***	
Observations	345,053											
Clusters	167,150											

Notes: Odds ratios (OR) and 95% Confidence Intervals (CI) are from multilevel logistic regressions, controlling for physician specialty, physician age category, primary office state, review submission year, and review word count. Estimated probabilities (EP) are obtained using the "margins" command in Stata. Physician-level random effects include variance of provider random intercept (σ^2) and its 95% CI, Intraclass Correlation Coefficient (ICC) and its 95% CI, and Likelihood Ratio Test (LRT; χ^2). *p<.05; **p<.01; ***p<.001.

© 2025 Madanay F et al. *JAMA Network Open*.

eTable 2. Odds ratios, estimated probabilities, and physician-level random effects of female vs male PCPs receiving any, positive, or negative interpersonal manner and technical competence comments

	Any interpersonal manner		Positive interpersonal manner		Negative interpersonal manner		Any technical competence		Positive technical competence		Negative technical competence	
	OR [95% CI]	EP (%)	OR [95% CI]	EP (%)	OR [95% CI]	EP (%)	OR [95% CI]	EP (%)	OR [95% CI]	EP (%)	OR [95% CI]	EP (%)
Female PCP	1.13*** [1.10, 1.16]	79.7	0.98 [0.96, 1.00]	60.6	1.22*** [1.18, 1.27]	19.0	1.00 [0.98, 1.03]	53.2	0.98 [0.96, 1.00]	41.3	1.08*** [1.04, 1.13]	12.1
Male PCP	1.00	77.8	1.00	61.1	1.00	16.7	1.00	53.1	1.00	41.8	1.00	11.4
Physician-level random effects												
σ^2	0.37 [0.33, 0.43]		0.58 [0.53, 0.63]		1.25 [1.14, 1.37]		0.26 [0.22, 0.30]		0.34 [0.30, 0.38]		1.27 [1.12, 1.43]	
ICC	0.10 [0.09, 0.12]		0.15 [0.14, 0.16]		0.28 [0.26, 0.29]		0.07 [0.06, 0.08]		0.09 [0.08, 0.10]		0.28 [0.25, 0.30]	
LRT (χ^2)	402.53***		1206.06***		1393.48***		325.59***		517.01***		685.04***	
Observations	169,267											
Clusters	131,018											

Notes: Odds ratios (OR) and 95% Confidence Intervals (CI) are from multilevel logistic regressions, controlling for physician age category, primary office state, review submission year, and review word count. Estimated probabilities (EP) are obtained using the “margins” command in Stata. Physician-level random effects include variance of provider random intercept (σ^2) and its 95% CI, Intraclass Correlation Coefficient (ICC) and its 95% CI, and Likelihood Ratio Test (LRT; χ^2). *p<.05; **p<.01; ***p<.001.

© 2025 Madanay F et al. *JAMA Network Open*.

eTable 3. Odds ratios, estimated probabilities, and physician-level random effects of female vs male surgeons receiving any, positive, or negative interpersonal manner and technical competence comments

	Any interpersonal manner		Positive interpersonal manner		Negative interpersonal manner		Any technical competence		Positive technical competence		Negative technical competence	
	OR [95% CI]	EP (%)	OR [95% CI]	EP (%)	OR [95% CI]	EP (%)	OR [95% CI]	EP (%)	OR [95% CI]	EP (%)	OR [95% CI]	EP (%)
Female Surgeon	1.35*** [1.29, 1.42]	74.8	1.30*** [1.24, 1.37]	63.6	1.01 [0.92, 1.11]	11.5	1.02 [0.97, 1.07]	72.7	1.04 [0.99, 1.09]	61.3	0.96 [0.89, 1.04]	11.3
Male Surgeon	1.00	69.2	1.00	58.0	1.00	11.4	1.00	72.4	1.00	60.5	1.00	11.6
Physician-level random effects												
σ^2	0.25 [0.23, 0.27]		0.40 [0.38, 0.42]		1.58 [1.50, 1.67]		0.21 [0.20, 0.24]		0.41 [0.39, 0.43]		0.99 [0.92, 1.05]	
ICC	0.07 [0.07, 0.08]		0.11 [0.10, 0.11]		0.32 [0.31, 0.34]		0.06 [0.06, 0.07]		0.11 [0.10, 0.12]		0.23 [0.22, 0.24]	
LRT (χ^2)	1500.76		3432.28***		5015.80***		768.09***		3244.82***		2575.69***	
Observations	175,786											
Clusters	36,132											

Notes: Odds ratios (OR) and 95% Confidence Intervals (CI) are from multilevel logistic regressions, controlling for physician age category, primary office state, review submission year, and review word count. Estimated probabilities (EP) are obtained using the "margins" command in Stata. Physician-level random effects include variance of provider random intercept (σ^2) and its 95% CI, Intraclass Correlation Coefficient (ICC) and its 95% CI, and Likelihood Ratio Test (LRT; χ^2). *p<.05; **p<.01; ***p<.001.

© 2025 Madanay F et al. *JAMA Network Open*.

eTable 4. Odds ratios, estimated probabilities, and physician-level random effects of female vs male PCPs receiving high-star ratings when patients comment on their interpersonal manner and technical competence

	OR [95% CI]	EP (%)
Positive interpersonal manner		
Female PCP	1.01 [0.91, 1.14]	91.3
Male PCP	1.00	92.9
Positive technical competence		
Female PCP	0.82* [0.70, 0.96]	84.4
Male PCP	1.00	87.1
Negative interpersonal manner		
Female PCP	0.62*** [0.53, 0.73]	13.2
Male PCP	1.00	21.0
Negative technical competence		
Female PCP	0.60*** [0.50, 0.73]	36.1
Male PCP	1.00	44.7
Physician-level random effects		
σ^2	0.13 [0.07, 0.25]	
ICC	0.04 [0.02, 0.07]	
LRT (χ^2)	10.90***	
Observations	169,267	
Clusters	131,018	

Notes: Odds ratios (OR) and 95% Confidence Intervals (CI) are from multilevel logistic regressions, controlling for physician age category, primary office state, review submission year, and review word count. Estimated probabilities (EP) are obtained using the "margins" command in Stata. Physician-level

© 2025 Madanay F et al. *JAMA Network Open*.

random effects include variance of provider random intercept (σ^2) and its 95% CI, Intraclass Correlation Coefficient (ICC) and its 95% CI, and Likelihood Ratio Test (LRT; χ^2). *p<.05; **p<.01; ***p<.001.

© 2025 Madanay F et al. *JAMA Network Open*.

eTable 5. Odds ratios, estimated probabilities, and physician-level random effects of female vs male surgeons receiving high-star ratings when patients comment on their interpersonal manner and technical competence

	OR [95% CI]	EP (%)
Positive interpersonal manner		
Female surgeon	1.09 [0.83, 1.43]	94.2
Male surgeon	1.00	95.3
Positive technical competence		
Female surgeon	1.06 [0.77, 1.45]	94.1
Male surgeon	1.00	94.6
Negative interpersonal manner		
Female surgeon	0.91 [0.64, 1.29]	38.8
Male surgeon	1.00	42.1
Negative technical competence		
Female surgeon	0.67** [0.50, 0.89]	44.2
Male surgeon	1.00	51.9
Physician-level random effects		
σ^2	0.67 [0.57, 0.78]	
ICC	0.17 [0.15, 0.19]	
LRT (χ^2)	288.55***	
Observations	175,786	
Clusters	36,132	

Notes: Odds ratios (OR) and 95% Confidence Intervals (CI) are from multilevel logistic regressions, controlling for physician age category, primary office state, review submission year, and review word count. Estimated probabilities (EP) are obtained using the “margins” command in Stata. Physician-level

© 2025 Madanay F et al. *JAMA Network Open*.

random effects include variance of provider random intercept (σ^2) and its 95% CI, Intraclass Correlation Coefficient (ICC) and its 95% CI, and Likelihood Ratio Test (LRT; χ^2). *p<.05; **p<.01; ***p<.001.

© 2025 Madanay F et al. *JAMA Network Open*.

eTable 6. Characteristics of physicians with and without written reviews

	Physicians with reviews N = 167,150	Physicians without reviews N = 85,000
Physician gender, No. (%)	167,150 (100)	85,000 (100)
Female	60,060 (35.9)	32,220 (37.9)
Male	107,090 (64.1)	52,780 (62.1)
Physician specialty, No. (%)	167,150 (100)	85,000 (100)
Primary care	131,018 (78.4)	73,607 (86.6)
Surgery	36,132 (21.6)	11,393 (13.4)
Physician age[a], M [SD]	55.16 [11.40]	58.67 [13.07]
Number of star ratings, No. (%)	2,147,770 (100)	292,477 (100)
Female PCP	563,946 (26.3)	100,591 (34.4)
Male PCP	861,409 (40.1)	147,725 (50.5)
Female surgeon	60,212 (2.8)	4,656 (1.6)
Male surgeon	662,203 (30.8)	39,505 (13.5)
Number of star ratings per physician, M [SD], range	12.85 [18.94], 1–1186	3.44 [3.82], 1–345
Female PCP	10.10 [11.87], 1–613	3.26 [3.32], 1–69
Male PCP	11.45 [13.76], 1–911	3.45 [3.53], 1–220
Female surgeon	14.18 [29.79], 1–1186	3.37 [3.32], 1–24
Male surgeon	20.77 [31.45], 1–1068	3.95 [5.89], 1–345

© 2025 Madanay F et al. *JAMA Network Open*.

Mean star rating, M [SD]	3.95 [0.97]	4.05 [1.26]
Female PCP	3.85 [1.04]	4.05 [1.27]
Male PCP	3.92 [0.98]	4.04 [1.27]
Female surgeon	4.25 [0.85]	4.29 [1.12]
Male surgeon	4.15 [0.81]	4.06 [1.24]

Notes: [a]Physician age was missing for n=15,406 (9.2%) physicians with reviews and for n=5,913 (7.0%) physicians without reviews. Age is conditional on non-missing.

© 2025 Madanay F et al. *JAMA Network Open*.
