# Supplementary material for: Physician Gender and Patient Perceptions of Interpersonal and Technical Skills in Online Reviews
Source: JAMA Netw Open. 2025 Feb 14;8(2):e2460018. doi: 10.1001/jamanetworkopen.2024.60018 (PMC11829228; doi:10.1001/jamanetworkopen.2024.60018)
Supplement: Supplement 2. — Data Sharing Statement [file jamanetwopen-e2460018-s002.pdf]

Data Sharing Statement

Madanay. Physician Gender and Patient Perceptions of Interpersonal and Technical Skills in Online Reviews. *JAMA Netw Open*. Published February 14, 2025.
doi:10.1001/jamanetworkopen.2024.60018

Data
Data available: No

Additional Information
Explanation for why data not available: As we are continuing to use the data for yet unpublished projects, data will be available only upon request.
